# Characterizing the clinical profile of mania without major depressive episodes: a systematic review and meta-analysis of factors associated with unipolar mania

**DOI:** 10.1017/S0033291723000831

**Published:** 2023-11

**Authors:** Francesco Bartoli, Christian Nasti, Dario Palpella, Susanna Piacenti, Maria Elisa Di Lella, Stefano Mauro, Luca Prestifilippo, Cristina Crocamo, Giuseppe Carrà

**Affiliations:** 1 Department of Medicine and Surgery, University of Milano-Bicocca, via Cadore 48, 20900 Monza, Italy; 2 Division of Psychiatry, University College London, Maple House 149, London W1T 7BN, UK

**Keywords:** Bipolar disorder, classification, meta-analysis, mood disorders, unipolar mania

## Abstract

**Background:**

The diagnostic concept of unipolar mania (UM), i.e. the lifetime occurrence of mania without major depressive episodes, remains a topic of debate despite the evidence accumulated in the last few years. We carried out a systematic review and meta-analysis of observational studies testing factors associated with UM as compared to bipolar disorder with a manic-depressive course (md-BD).

**Methods:**

Studies indexed up to July 2022 in main electronic databases were searched. Random-effects meta-analyses of the association between UM and relevant correlates yielded odds ratio (OR) or standardized mean difference (SMD), with 95% confidence intervals (CIs).

**Results:**

Based on data from 21 studies, factors positively or negatively associated with UM, as compared to md-BD, were: male gender (OR 1.47; 95% CI 1.11–1.94); age at onset (SMD −0.25; 95% CI −0.46 to −0.04); number of hospitalizations (SMD 0.53; 95% CI 0.21–0.84); family history of depression (OR 0.55; 95% CI 0.36–0.85); suicide attempts (OR 0.25; 95% CI 0.19–0.34); comorbid anxiety disorders (OR 0.35; 95% CI 0.26–0.49); psychotic features (OR 2.16; 95% CI 1.55–3.00); hyperthymic temperament (OR 1.99; 95% CI 1.17–3.40). The quality of evidence for the association with previous suicide attempts was high, moderate for anxiety disorders and psychotic features, and low or very low for other correlates.

**Conclusions:**

Despite the heterogeneous quality of evidence, this work supports the hypothesis that UM might represent a distinctive diagnostic construct, with peculiar clinical correlates. Additional research is needed to better differentiate UM in the context of affective disorders, favouring personalized care approaches.

## Introduction

During 1960s, pioneering research highlighted that manic-depressive illness, as conceptualized by Emil Kraepelin (1856–1926), is not nosologically homogeneous, stressing the differences between the unipolar and bipolar course of affective disorders in terms of genetics, premorbid personality, and clinical outcomes (Angst & Marneros, 2001). Bipolar disorder (BD) is a multidimensional condition which includes a multitude of clinical subtypes (Ghaemi et al., 2022; McIntyre et al., 2022) and might be based on different neurobiological underpinnings (Han, De Berardis, Fornaro, & Kim, 2019; Sepede et al., 2020). Over the years, the diagnostic concept of unipolar mania (UM), i.e. the lifetime occurrence of mania without major depressive episodes, has been proposed and widely debated (Angst, 1978; Angst & Grobler, 2015; Nurnberger, Roose, Dunner, & Fieve, 1979; Perugi, Passino, Toni, Maremmani, & Angst, 2007; Pfohl, Vasquez, & Nasrallah, 1982; Shulman & Tohen, 1994). The modern criteria of the Diagnostic and Statistical Manual of Mental Disorders (DSM) (American Psychiatric Association, 1994, 2013) agreed that type-1 BD can be diagnosed on the basis of the occurrence of just a single manic episode, without providing any differentiation between UM and BD with major depressive episodes (md-BD) (Angst, 2015; Ghaemi et al., 2022). Indeed, even though the role of UM as a separate diagnostic entity has been claimed (Angst & Grobler, 2015; Yazıcı, 2014), its clinical characterization is not well defined so far and the research literature on pure mania remains sparse. Data from the U.S. National Epidemiologic Survey on Alcohol and Related Conditions estimated that the prevalence of UM among people with BD range from 5.0% to 7.2%, with only a partial diagnostic stability, considering that about one out of five people develop md-BD within 3 years (Baek, Eisner, & Nierenberg, 2014).

Exploring the hypothesis of UM as an independent clinical entity, previous studies have preliminarily suggested that it would differ from md-BD, in terms of several clinical characteristics, including disease onset, recurrences, premorbid temperament, and comorbid conditions (Angst & Grobler, 2015). However, findings in this field remain sparse (e.g. Chang et al., 2022; Sangha et al., 2022; Stokes et al., 2020) and no systematic analyses of individual characteristics associated with UM are available so far. Identifying correlates of UM could be useful to clarify if these subjects might represent a subpopulation with specific clinical profiles and unmet care needs, requiring personalized treatments, as compared with those suffering from md-BD (Angst & Grobler, 2015; Mehta, 2014; Yazıcı, 2014). To shed light on this topic, we performed a systematic review and meta-analysis of observational studies aimed at identifying sociodemographic and clinical correlates of UM, also assessing the generated quality of evidence in terms of strength, precision, consistency, and risk of bias.

## Methods

### Study design and protocol

The current systematic review and meta-analysis is based on the Preferred Reporting Items for Systematic Reviews and Meta-Analyses (PRISMA) 2020 statement (Page et al., 2021). The study protocol registration was completed in Open Science Framework registries on 11 July 2022 (doi: 10.17605/OSF.IO/95RDV).

### Eligibility criteria

We included any observational studies comparing UM and md-BD on one or more sociodemographic or clinical characteristics. To be considered, studies had to include at least 10 individuals in each group. We excluded studies (a) not providing information on UM, (b) without relevant md-BD controls, (c) involving individuals with >10% of non-BD diagnoses, such as schizoaffective disorders, (d) including samples with a mean age <18 years, (e) not providing sufficient data, and (f) being published before the release date of DSM-IV (American Psychiatric Association, 1994). We excluded also data deriving from the same sample, to avoid duplicate results, and scientific reports not undergoing peer-review process, such as conference abstracts, dissertations, and grey literature.

### Search strategy and study selection

We searched Embase, Ovid MEDLINE, and APA PsycInfo databases (via Ovid) for articles indexed up to July 2022, without language restrictions. The full search strategy is reported in online Supplementary File 1. We carried out an additional, post hoc, non-systematic search on Google Scholar to check whether additional studies were retrievable. We performed also a manual search of the reference lists of four relevant reviews (Angst, 2015; Angst & Grobler, 2015; Dondé, Lepetit, & Lavigne, 2019; Yazıcı, 2014). We completed the preliminary screening based on titles and abstracts. Full texts were then retrieved to assess studies according to inclusion criteria for final eligibility. Disagreements concerning suitability for inclusion were resolved by discussion and consensus, involving all authors.

### Data extraction

We used a standard template to extract key information for all eligible studies: year of publication; country; setting; inclusion criteria; sample size, mean age, and sex proportion; definition of UM; methods to assess UM and md-BD; and sociodemographic and clinical correlates of UM. If needed the corresponding authors were contacted to obtain relevant data. Six authors independently extracted data for blind check of accuracy.

### Risk of bias assessment

First, we evaluated the risk of selection bias by checking whether UM and md-BD groups were comparable in terms of age and illness duration, respectively. We considered as acceptable a non-statistical difference (*p* > 0.05) or a difference of no more than 3 years between groups. Second, we assessed the representativeness of included samples, verifying whether participants were selected from special populations in terms of age, gender, or clinical characteristics. Finally, we evaluated the included studies for potential sources of misclassification bias assessing the criteria used to diagnose UM. We considered appropriate a definition of UM as the lifetime occurrence of at least three manic episodes without depressive episodes during a period of observation of at least 4 years (Baek et al., 2014).

### Data analysis

Meta-analyses were performed for each correlate with data available from at least five different studies or samples. Meta-analyses of the association between UM and relevant correlates were based on odds ratio (OR) with 95% confidence interval (CI) and standardized mean difference (SMD) with 95% CI, for categorical and continuous variables, respectively. Pooled estimates were obtained by weighting each study according to a random-effects model. Heterogeneity across studies was evaluated according to standard cut-offs for *I*^2^ statistics (Higgins, Thompson, Deeks, & Altman, 2003). Publication bias was assessed using Egger's test for correlates with data available from at least 10 studies (Sterne, Egger, & Moher, 2008). We used the trim-and-fill method (Duval & Tweedie, 2000) for analyses showing an Egger's test *p*-value <0.10. Sensitivity analyses of between-study heterogeneity were performed for statistically significant (*p* < 0.05), but inconsistent (*I*^2^ > 50%), estimates, based on at least 10 studies. We left out the minimum number of studies needed to reach an *I*^2^ value below the predefined threshold of 50% (Patsopoulos, Evangelou, & Ioannidis, 2008). Finally, additional sensitivity analyses were carried out, to estimate the effect of risk of bias, sequentially excluding studies with low quality in each of the considered items, i.e. comparability, representativeness, and UM definition. Data analyses were performed using Stata statistical software, Release 17 (StataCorp LLC, College Station, TX). Forest plots were generated using OpenMeta[Analyst] (Wallace, Schmid, Lau, & Trikalinos, 2009).

### Grading of the evidence

We used GRADE items (Schünemann et al., 2022a), adapted for non-interventional observational studies, to classify the quality of evidence as high, moderate, low or very low, for each correlate showing a statistically significant estimate (*p* < 0.05).

First, we evaluated the ‘magnitude of the effect’ according to the cut-offs for SMD magnitude (0.2 small, 0.5 medium, 0.8 large) (Schünemann et al., 2022b). In order to estimate their magnitude of effect, we converted ORs into SMDs dividing the relevant ln(OR) by 1.81 (Chinn, 2000). We downgraded the quality of evidence by one level if the magnitude was small (SMD < 0.35).

Second, we assessed the effect of ‘risk of bias’ by verifying whether sensitivity analyses – excluding studies with lower quality in each evaluated item (comparability, representativeness, and UM definition) – were consistent with the findings of the overall analysis. We downgraded the quality of evidence by one level if at least one sensitivity analysis yielded a non-significant estimate for the association between the tested variables and UM.

Third, we evaluated the ‘precision’ of findings by checking the width of the 95% CI, downgrading the quality of evidence by one level if UM correlates were based on a width of their 95% CI ⩾0.4.

In addition, we assessed the ‘consistency’ of findings according to the *I*^2^ value. We downgraded by one level the quality of evidence if inconsistency was estimated (*I*^2^ ⩾ 50%) and the between-study heterogeneity sensitivity analysis was not significant (*p* > 0.05).

Finally, we estimated the risk of ‘publication bias’, downgrading the quality of evidence by one level if (*a*) less than 10 studies were included or (*b*) Egger's test *p*-value was <0.10 and the trim-and-fill method did not show an association between the tested variable and UM.

## Results

### Study selection and characteristics

Our systematic search generated 5574 articles (2584 from Embase, 1362 from Medline, 1628 from PsycInfo), reduced to 3287 after deduplication. Among them, 79 were identified as potentially eligible after the screening by titles and abstracts, including one study retrieved from the additional check of the reference list of a recent review (Angst & Grobler, 2015). After the full-text revision, 58 studies were excluded.

**Fig. 1.**
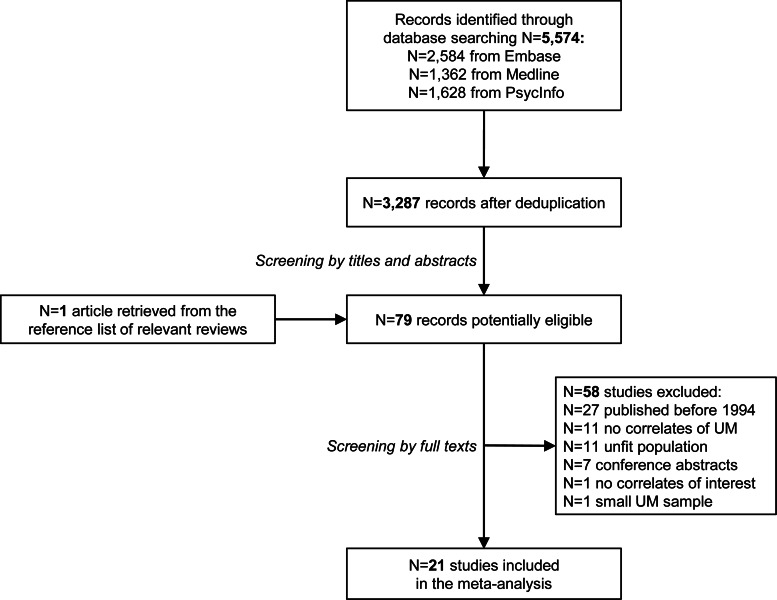
Flowchart of study selection process.

Twenty-one studies met the eligibility criteria and were included in the meta-analysis (Aghanwa, 2001; Akarsu et al., 2012; Amamou et al., 2018; Andrade-Nascimento, Miranda-Scippa, Nery-Fernandes, Kapczinski, & Quarantini, 2011; Angst, Gerber-Werder, Zuberbühler, & Gamma, 2004; Angst et al., 2019; Beesdo et al., 2009; Chang et al., 2022; Dakhlaoui, Essafi, & Haffani, 2008; Gorgulu, Uluturk, & Palabiyik, 2021; Grobler, Roos, & Bekker, 2014; Grover et al., 2021; Mittal, Mehta, Solanki, Swami, & Meena, 2013; Perugi et al., 2007; Rajkumar, 2016; Sangha et al., 2022; Sonkurt, Altınöz, Danışman Sonkurt, & Köşger, 2021; Stokes et al., 2020; Subramanian, Kattimani, Rajkumar, Bharadwaj, & Sarkar, 2016; Yazıcı & Çakır, 2012; Yazici et al., 2002). The flowchart with details on screening and study selection process is reported in Fig. 1.

The main characteristics of the included studies are reported in Table 1. Some of them encompassed multiple samples: the study of Angst et al. (2019) combined seven population-based studies whose data were merged for inclusion in our meta-analyses; the study of Chang et al. (2022) included two different cohorts, i.e. the Genomic Research and Epidemiological Studies for Affective Disorders in Taiwan and the Psychiatric Inpatients Medical Claim, but data from the latter were not used in our meta-analysis, since a high proportion of UM diagnoses were later re-assessed mainly as psychotic disorders; the study of Stokes et al. (2020) included two cohorts, one from France and the other from the UK, and relevant data were managed separately in our meta-analyses. For the study by Beesdo et al. (2009), not providing raw data, information on different variables associated with UM were retrieved from Angst et al. (2019). Two studies (Rajkumar, 2016; Subramanian et al. 2016) had a partial overlap of samples. We prioritized data from Rajkumar (2016), despite its smaller sample size, since data from Subramanian et al. (2016) were unpublished (provided by the corresponding author). We thus used for our meta-analyses only those variables from Subramanian et al. (2016) that were not included in the study by Rajkumar (2016).

**Table 1.** Characteristics of included studies

Study	Country	Setting	Sample size, *N*	Age mean or median	Males (%)	UM, *N*	md-BD, *N*	UM	
								ME	YO
Aghanwa (2001)	Republic of Fiji	Inpatient	82	NR	52.4	51	31	3	4
Akarsu et al. (2012)	Turkey	Inpatient	108	31.0	78.7	16	92	4	NR
Amamou et al. (2018)	Tunisia	Inpatient	173	43.3	68.2	101	72	3	NR
Andrade-Nascimento et al. (2011)	Brazil	Outpatient	298	41.6	31.9	16	282	NR	15
Angst et al. (2004)	Switzerland	Inpatient	160	63.4	40.6	30	130	1	17.6
Angst et al. (2019)	Multiple countries	Mixed	432	35.5	43.1	109	323	1	NR
Beesdo et al. (2009)	Germany	Community	84	25.3	50.0	33	51	1	7-10
Chang et al. (2022) – GREAT cohort	Taiwan	Inpatient	1015	40.0	44.9	131	884	1	NR
Dakhlaoui et al. (2008)	Tunisia	Inpatient	72	36.2	58.3	47	25	2	5
Gorgulu et al. (2021)	Turkey	Inpatient	80	42.3	45.0	38	42	4	4
Grobler et al. (2014)	South Africa	Inpatient	103	36.6	44.7	59	44	3	NR
Grover et al. (2021)	India	Outpatient	714	45.6	NR	42	672	4	5
Mittal et al. (2013)	India	Outpatient	60	32.5	86.7	30	30	2	NR
Perugi et al. (2007)	Italy	Inpatient	87	44.2	44.8	19	68	3	10
Rajkumar (2016)	India	Inpatient	150	31.6	36.4	38	28	2	NR
Sangha et al. (2022)	UK	Community	6030	52.5	42.8	1200	4830	NR	NR
Sonkurt et al. (2021)	Turkey	Outpatient	37	42.6	67.6	18	19	4	4
Stokes et al. (2020)	France	Inpatient and outpatient	392	41.1	45.9	13	379	3	10
	UK		34	49	58.8	17	17		
Subramanian et al. (2016)	India	Inpatient	150	37.8	48.0	79	71	NR	3
Yazıcı and Çakır (2012)	Turkey	Outpatient	121	44.8	39.7	34	87	4	4
Yazici et al. (2002)	Turkey	Outpatient	272	39	38.2	48	224	4	4

UM, unipolar mania; md-BD, bipolar disorder with major depressive episodes; ME, number of manic episodes required for UM diagnosis; YO, minimum years of observation without depressive episodes required; NR, unclear or not reported.

### Risk of bias assessment

In terms of age comparability between UM and md-BD, 10 studies met the quality criterion (Akarsu et al., 2012; Beesdo et al., 2009; Chang et al., 2022; Gorgulu et al., 2021; Perugi et al., 2007; Rajkumar, 2016; Sangha et al., 2022; Sonkurt et al., 2021; Stokes et al., 2020, UK cohort; Subramanian et al., 2016). On the contrary, illness duration comparability was warranted by the majority of studies, with just a few with unclear data (Grobler et al., 2014; Grover et al., 2021; Sangha et al., 2022; Yazıcı & Çakır, 2012) or a mean difference over 3 years between groups (Aghanwa, 2001; Andrade-Nascimento et al., 2011; Perugi et al., 2007; Sonkurt et al., 2021; Stokes et al., 2020, France cohort). Most of the included studies were sufficiently representative, apart from one involving only subjects with disease onset during adolescence (Beesdo et al., 2009) and another which sampled also people with non-BD diagnoses (Grobler et al., 2014), respectively. Finally, in terms of UM diagnosis, 11 included studies considered the threshold of at least three lifetime manic episodes (Aghanwa, 2001; Akarsu et al., 2012; Amamou et al., 2018; Gorgulu et al., 2021; Grobler et al., 2014; Grover et al., 2021; Perugi et al., 2007; Sonkurt et al., 2021; Stokes et al., 2020; Yazıcı & Çakır, 2012; Yazici et al., 2002) and 12 the minimum period of observation of 4 years to define UM (Aghanwa, 2001; Andrade-Nascimento et al., 2011; Angst et al., 2004; Beesdo et al., 2009; Dakhlaoui et al., 2008; Gorgulu et al., 2021; Grover et al., 2021; Perugi et al., 2007; Sonkurt et al., 2021; Stokes et al., 2020; Yazıcı & Çakır, 2012; Yazici et al., 2002). The risk of bias assessment of included studies is reported in online Supplementary File 2.

### Factors associated with UM: meta-analyses

Twenty different variables had data from at least five studies or samples, and were thus meta-analysed. They were grouped into five main categories, i.e. sociodemographic characteristics, clinical features, comorbidities, family history of mental disorders, and psychopharmacological treatment. The summary of findings is reported in Table 2.

**Table 2.** Sociodemographic and clinical correlates of UM: summary of findings

Variables	*k*	*N*	Effect size (95% CI)	*p*	*I*^2^ (%)
*Sociodemographic characteristics*					
Male gender	17	9462	**OR 1.47 (1.11–1.94)**	**0.007**	66.0
In a relationship	8	1783	OR 1.02 (0.79–1.33)	0.86	0
Unemployed	8	1017	OR 1.02 (0.75–1.38)	0.90	0
Higher education	8	7797	OR 0.98 (0.72–1.32)	0.88	40.4
*Clinical features*					
Age at disease onset	12	2716	**SMD −0.25 (−0.46 to −0.04)**	**0.020**	67.8
Number of mood episodes	10	1290	SMD −0.24 (−0.53 to 0.04)	0.09	71.9
Number of hospitalizations	6	1172	**SMD 0.53 (0.21–0.84)**	**<0.001**	65.5
Suicide attempts	15	3947	**OR 0.25 (0.19–0.34)**	**<0.001**	0
Rapid cycling course	7	1390	OR 0.68 (0.31–1.51)	0.34	7.0
Psychotic features	10	2631	**OR 2.16 (1.55–3.00)**	**<0.001**	27.4
Hyperthymic temperament	6	696	**OR 1.99 (1.17–3.40)**	**0.012**	17.7
*Comorbid mental disorders*					
Anxiety disorders	7	1786	**OR 0.35 (0.26–0.49)**	**<0.001**	0
Alcohol-use disorders	6	1497	OR 1.32 (0.84–2.08)	0.23	36.8
Substance-use disorders	5	949	OR 0.86 (0.46–1.62)	0.64	43.7
*Family history of mental disorders*					
Bipolar disorder	8	1078	OR 0.89 (0.63–1.25)	0.49	0
Major depressive disorder	6	923	**OR 0.55 (0.36–0.85)**	**0.006**	0
Alcohol-use disorders	5	504	OR 1.04 (0.64–1.68)	0.88	9.1
*Treatment-related variables*					
Lithium	7	1005	OR 0.81 (0.49–1.35)	0.42	37.6
Valproate	6	835	OR 1.39 (0.72–2.68)	0.33	60.2
Atypical antipsychotics	4^a^	711	OR 0.82 (0.32–2.12)	0.68	43.8

*k*, number of included studies; *N*, sample size; CI, confidence interval; OR, odds ratio; SMD, standardized mean difference. Statistically significant results are reported in bold.

a Four studies including five independent samples.

#### Sociodemographic characteristics

Meta-analytic data showed that individuals with UM were more likely to be males (*k* = 17; OR 1.47; 95% CI 1.11–1.94), with moderate-high heterogeneity across studies (*I*^2^ = 66.0%). The relevant sensitivity analysis, excluding one study accounting for almost all inconsistency (Sangha et al., 2022), confirmed the overall finding (*k* = 16; OR 1.38; 95% CI 1.08–1.77; *I*^2^ = 31.2%). The Egger's test estimated a significant risk of publication bias (*p* = 0.004). However, the trim-and-fill method confirmed the overall analysis showing an OR of 1.85 (95% CI 1.41–2.42). No differences between UM and md-BD were estimated for other sociodemographic characteristics (marital status, employment, and education). Forest plots are shown in online Supplementary Files 3–6.

#### Clinical features

Subjects with UM had a younger mean age at disease onset (*k* = 12; SMD −0.25; 95% CI −0.46 to −0.04) as compared with those with md-BD. However, results were inconsistent (*I*^2^ = 67.8%). The related sequential sensitivity analysis confirmed the overall estimate, after excluding two studies (Chang et al., 2022; Perugi et al., 2007) accounting for most of the heterogeneity (*k* = 10; SMD −0.35; 95% CI −0.53 to −0.16; *I*^2^ = 41.0%). Egger's test for publication bias was statistically significant (*p* = 0.048), but the trim-and-fill method corroborated the overall analysis (SMD −0.35; 95% CI −0.60 to −0.11). In addition, we estimated that participants with UM, as compared with those suffering from md-BD, had a higher number of lifetime hospitalizations (*k* = 6; SMD 0.53; 95% CI 0.21–0.84; *I*^2^ = 65.5%). The between-study heterogeneity sensitivity analysis excluding one study (Amamou et al., 2018), confirmed the results of the overall analysis (*k* = 5; SMD 0.67; 95% CI 0.45–0.89; *I*^2^ = 0%). Moreover, UM individuals had lower rates of previous suicide attempts (*k* = 15; OR 0.25; 95% CI 0.19–0.34; *I*^2^ = 0%; Egger's *p* = 0.37) were more likely to report psychotic features (*k* = 10; OR 2.16; 95% CI 1.55–3.00; *I*^2^ = 27.4%; Egger's *p* = 0.43) and hyperthymic temperament (*k* = 6; OR 1.99; 95% CI 1.17–3.40; *I*^2^ = 17.7%). Finally, no differences between UM and md-BD were estimated for mood episodes and a rapid cycling course. Forest plots are shown in online Supplementary Files 7–13.

#### Comorbidities

Participants with UM were less likely than those with md-BD to suffer from comorbid anxiety disorders (*k* = 7; OR 0.35; 95% CI 0.26–0.49; *I*^2^ = 0%). No differences in alcohol (*k* = 6; OR 1.32; 95% CI 0.84–2.08; *I*^2^ = 36.8%) and substance (*k* = 5; OR 0.86; 95% CI 0.46–1.62; *I*^2^ = 43.7%) use disorders were estimated. Forest plots are displayed in online Supplementary Files 14–16.

#### Family history of mental disorders

Subjects with UM were less likely to report a family history of depression than those with md-BD (*k* = 6; OR 0.55; 95% CI 0.36–0.85; *I*^2^ = 0%), while no differences were estimated for family history of BD (*k* = 8; OR 0.89; 95% CI 0.63–1.25; *I*^2^ = 0.0%) and alcohol-use disorders (*k* = 5; OR 1.04; 95% CI 0.64–1.68; *I*^2^ = 9.1%). Forest plots are reported in online Supplementary Files 17–19.

#### Psychopharmacological treatment

Meta-analyses showed that the prescription of lithium (*k* = 7; OR 0.81; 95% CI 0.49–1.35; *I*^2^ = 37.6%), valproate (*k* = 6; OR 1.39; 95% CI 0.72–2.68; *I*^2^ = 60.2%), and atypical antipsychotics (based on four studies and five samples; OR 0.82; 95% CI 0.32–2.12; *I*^2^ = 43.8%) did not significantly differ between subjects with UM and those with md-BD. Forest plots are shown in online Supplementary Files 20–22.

Results of all quality-based sensitivity analyses are reported in online Supplementary File 23.

### Grading of the evidence

A high quality of evidence was estimated just for one correlate, i.e. previous suicide attempts, based on a large magnitude, precision, and consistency of the effect, without any influence of publication bias and quality of included studies. Findings on psychotic features and anxiety disorders were based on a moderate quality of evidence, considering some quality issues of included studies and publication bias, respectively. The body of evidence for the remaining variables (number of hospitalizations, age at onset, hyperthymic temperament, and family history of depression) was deemed of low or very low quality, considering several downgrading on a number of items. The overall assessment of the quality of evidence is shown in Table 3.

**Table 3.** Grading of the evidence

Variables	Magnitude	Risk of bias	Imprecision	Inconsistency	Publication bias	Quality of evidence
Male gender	Small	Yes	No	No	No	Low
Age at disease onset	Small	Yes	Yes	No	No	Very low
Number of hospitalizations	Medium	Yes	Yes	No	Yes	Very low
Suicide attempts	Large	No	No	No	No	High
Psychotic features	Medium	Yes	No	No	No	Moderate
Hyperthymic temperament	Medium	Yes	Yes	No	Yes	Very low
Anxiety disorders	Medium	No	No	No	Yes	Moderate
Family history of depression	Small	Yes	Yes	No	Yes	Very low

## Discussion

### Summary of findings

To our knowledge, this is the first systematic review and meta-analysis aimed at identifying sociodemographic and clinical characteristics of UM as compared with md-BD. Based on data from 21 observational studies, we could estimate the relationship between UM and 20 potential correlates. Among them, eight were found to be associated with UM, with a variable effect magnitude. Study participants with UM, as compared to those with md-BD, were more likely to be males, with a younger age at disease onset. In addition, UM was associated with higher rates of hospitalizations, psychotic features, and hyperthymic temperament. Finally, participants with UM were less likely to have a history of depression, to suffer from comorbid anxiety disorders, and to report previous suicide attempts. On the contrary, it is worth mentioning that, despite the strict relationship between mania and addictive behaviours (e.g. Messer, Lammers, Müller-Siecheneder, Schmidt, & Latifi, 2017), no differences have been estimated under comorbid conditions, such as alcohol- and substance-use disorders, possibly due to their high rates in md-BD as well (Carrà et al., 2015; Hunt, Malhi, Cleary, Lai, & Sitharthan, 2016). As a whole, considering the significant differences between UM and md-BD especially in terms of non-modifiable risk factors (gender, age at onset, temperament, and family history of depression), our findings seem to support the hypothesis that UM might represent an independent subset of patients with peculiar clinical features (Angst & Grobler, 2015; Yazıcı, 2014). Nonetheless, considering the differential quality of evidence regarding the various correlates, the interpretation of our findings requires caution.

Indeed, the majority of characteristics tested in our systematic review and meta-analysis, namely sex, age at onset, number of hospitalizations, hyperthymic temperament, and family history of depression, were based on a low or very low quality of evidence. Relevant estimates were affected by some issues, in terms of poor quality of included studies, imprecision of the effect, and uncertainty in terms of publication bias, all significantly limiting the robustness of these meta-analytic findings. On the contrary, a high or moderate quality of evidence, according to the GRADE items, was appraised for the lower rates of suicide attempts in UM, the increased likelihood of psychotic features, and the lower frequency of comorbid anxiety disorders. These findings are not surprising, considering that depressive episodes, absent in UM by definition, might account for a large proportion of suicidal risk in BD (e.g. Gonda et al., 2012; Marangell et al., 2006), and might be associated with psychotic features less frequently than manic episodes (e.g. van Bergen et al., 2019). Similarly, comorbid anxiety disorders, such as generalized anxiety and panic disorders, are more likely to co-occur with, and negatively impact on, bipolar depression (e.g. Tohen et al., 2007).

### Clinical and research implications

Important implications and issues from our findings need to be considered before any conclusion can be drawn on UM as a distinct subgroup within BD. First, considering that the current diagnostic classifications do not differentiate between UM and md-BD (American Psychiatric Association, 2013), additional research is required for a better contextualization of UM within the bipolar and related disorders group. To fill this gap, solid recommendations are needed to define the number of manic episodes, the inclusion or exclusion of minor or sub-threshold depressive episodes, the role of mixed features, and the minimum length of observation required to make a diagnosis of UM. Special attention deserves the role of temperaments in the context of mood spectrum (Ghaemi et al., 2022), since individuals with hyperthymic temperaments, possibly due to high level of sensation seeking, seem more likely to develop manic episodes without depression as consistent with our findings.

Moreover, the lack of a nosological characterization of UM could hamper the efforts of research to determine also its potentially distinct aetiology (Angst et al., 2019). Consistently, the clinical distinction between UM and md-BD may benefit, along with the course of the illness, from further insight on neurobiological correlates of depression and mania (Abé et al., 2015; Passos, Mwangi, Vieta, Berk, & Kapczinski, 2016). Indeed, it remains unclear if specific neurobiological underpinnings might support the clinical differentiation between UM and md-BD we could uncover in this meta-analysis (Schmitt & Falkai, 2015). In particular, based on the evidence hypothesizing a potential role of inflammation in BD (e.g. Misiak et al., 2020), preliminary data investigating peripheral inflammatory markers have shown some differences between UM and md-BD in terms of C-reactive protein and interleukin-6 levels (Gorgulu et al., 2021). Moreover, it would be useful to clarify if other theoretical models hypothesized for the manic-depressive cycle of BD, involving neurotransmitter systems, neurotrophic factors, stress axis activity, chronobiology, oxidative stress, and mitochondrial dysfunctions, might be valid also for UM (e.g. Bartoli et al., 2021; Gonzalez, 2014; Sigitova, Fišar, Hroudová, Cikánková, & Raboch, 2017).

In addition, the diagnostic stability of UM over time remains a critical issue, considering the unclear likelihood of conversion to md-BD. The few available prospective studies have generated mixed findings with heterogenous conversion rates (e.g. Baek et al., 2014; Solomon et al., 2003). Nonetheless, it has been suggested that the diagnostic stability of UM would improve, if higher number of manic episodes would be required for its diagnosis (Angst & Grobler, 2015). Clearly, further longitudinal data are required to clarify this issue.

Finally, additional research is needed on potential treatments targeting UM. Indeed, although we could not estimate differences in prescription of lithium, valproate, and atypical antipsychotics, it is likely that the management of UM might differ from standard care of md-BD, given the lower likelihood of suicide attempts and of co-occurring anxiety disorders, and the higher number of hospitalizations. Nonetheless, no specific recommendations for treating UM in routine clinical practice are available so far. Some authors argued that the absence of depressive episodes would make the pharmacological prophylaxis of UM less complex than for md-BD (Angst et al., 2019). It is likely that lithium, the underused gold-standard treatment for BD (Bartoli, 2023), might represent the first-choice option for UM considering its efficacy in preventing manic episodes (Severus et al., 2014). In addition, considering the increased rates of concurrent psychotic features in UM and the effectiveness of second-generation antipsychotics in treating mania (Kishi et al., 2022), it can be hypothesized a more prominent role for these drugs in UM, especially in their long-acting formulations (Bartoli et al., 2022). For the same reason, non-pharmacological interventions including psychotherapeutic approaches with proven effectiveness for md-BD (e.g. Fiorillo et al., 2015; Miklowitz et al. 2021; Reinares, Sánchez-Moreno, & Fountoulakis, 2014) should be investigated also in people with UM.

### Limitations

The findings of this systematic review and meta-analysis should be interpreted with caution considering some limitations. First, since this work tested the cross-sectional associations of UM with sociodemographic and clinical correlates, we cannot draw any causal inference. Second, we need to consider the methodological inconsistency across studies in terms of study design, sample size, inclusion criteria, methods to assess single correlates, and UM definition among others. In particular, we found a high variability across studies in terms of follow-up and number of manic episodes required to define UM, with only a few studies using appropriate definitions of UM. Third, due to the descriptive and observational nature of included studies, we should take into account some potential risk of reporting bias. Although we estimated low probability of publication bias for several variables, we cannot rule out that unpublished data may have at least partially influenced the meta-analytic estimates for other correlates. Finally, data on several characteristics were available only from a limited number of studies, narrowing the relevant precision of estimates. Similarly, other meaningful correlates could not be explored due to the lack of sufficient data from eligible studies, thus preventing a more comprehensive assessment. For instance, few studies tested important descriptive elements that may influence the course of both UM and md-BD, such as the occurrence of mixed features (Solé, Garriga, Valentí, & Vieta, 2017; Verdolini et al., 2018) and comorbid attention-deficit/hyperactivity disorder (Bartoli et al., 2023; Brancati, Perugi, Milone, Masi, & Sesso, 2021). In particular, even though no differences were found between UM and md-BD in terms of substance-use disorders, the role of specific drugs, such as cannabis and cocaine (Bartoli, Crocamo, & Carrà, 2019; Gibbs et al., 2015; Lalli, Brouillette, Kapczinski, & de Azevedo Cardoso, 2021), should be clarified. In addition, key characteristics of UM have been highlighted in terms of personality traits and chronotype, including a higher proportion of ‘morningness’, better sleep quality, higher extraversion, lower neuroticism, and less avoidance personality traits (Chang et al., 2022). Additional studies are required to confirm these potential differences between UM and md-BD.

## Conclusions

As a whole, our findings seem to support at least partially the hypothesis that UM might represent a distinctive diagnostic entity, with peculiar clinical correlates. It is likely that treatment strategies for UM might be different from those used for BD with a standard manic-depressive course. Additional research is needed to substantiate the diagnostic independence of UM in the context of affective disorders, to delineate its epidemiological burden, and to identify relevant effective approaches for a personalized care.

## Supporting information

Bartoli et al. supplementary material 1 Bartoli et al. supplementary material

Bartoli et al. supplementary material 2 Bartoli et al. supplementary material

Bartoli et al. supplementary material 3 Bartoli et al. supplementary material

Bartoli et al. supplementary material 4 Bartoli et al. supplementary material

## Supplementary material

The supplementary material for this article can be found at https://doi.org/10.1017/S0033291723000831.
