# Supplementary material for: Characterizing the clinical profile of mania without major depressive episodes: a systematic review and meta-analysis of factors associated with unipolar mania
Source: Psychol Med. 2023 Apr 5;53(15):7277–86. doi: 10.1017/S0033291723000831 (PMC10719688; doi:10.1017/S0033291723000831)
Supplement: Bartoli et al. supplementary material 1 — Bartoli et al. supplementary material [file S0033291723000831sup001.docx]

# **Supplementary File 1. Full search strategy via Ovid**

Search date: 4^th^ July 2022.

Publication date limits: none.

Language restrictions: none.

Database: Embase <1974 to 2022 July 01>, Ovid MEDLINE(R) ALL <1946 to July 01, 2022>, APA PsycInfo <1806 to June Week 3 2022>

Search Strategy:

1     mania.mp. (56,994)

2     manic.mp. (47,658)

3     unipolar*.mp. (35,683)

4     (bipolar adj2 "1").mp. (1,759)

5     (bipolar adj2 I).mp. (17,721)

6     1 or 2 (84,593)

7     4 or 5 (19,143)

8     3 and 6 (4,765)

9     3 and 7 (1,521)

10   8 or 9 (5,574)
