# Supplementary material for: Characterizing the clinical profile of mania without major depressive episodes: a systematic review and meta-analysis of factors associated with unipolar mania
Source: Psychol Med. 2023 Apr 5;53(15):7277–86. doi: 10.1017/S0033291723000831 (PMC10719688; doi:10.1017/S0033291723000831)
Supplement: Bartoli et al. supplementary material 2 — Bartoli et al. supplementary material [file S0033291723000831sup002.docx]

**Supplementary File 23. Quality-based sensitivity analyses**

| **Variables** | **Age**  **comparability** | | **Illness duration comparability** | | **Sample**  **representativeness** | | **Number of**  **manic episodes** | | **Observation ≥4 yrs** | |
| --- | --- | --- | --- | --- | --- | --- | --- | --- | --- | --- |
|  | **k** | **Effect size (95%CI); p-value** | **k** | **Effect size (95%CI); p-value** | **k** | **Effect size (95%CI); p-value** | **k** | **Effect size (95%CI); p-value** | **k** | **Effect size (95%CI); p-value** |
| Male gender | 7 | OR = 1.41 (0.87 to 2.30); p = 0.16 | 10 | OR = 1.40 (1.10 to 1.78); **p = 0.005** | 15 | OR = 1.36 (1.00 to 1.85); p = 0.05 | 8 | OR = 1.37 (0.86 to 2.18); p = 0.18 | 10 | OR = 1.45 (1.02 to 2.05); **p = 0.036** |
| Age at disease onset | 7 | SMD = -0.11 (-0.38 to 0.17); p = 0.44 | 8 | SMD = -0.20 (-0.42 to 0.02); p = 0.08 | – | – | 8 | SMD = -0.28 (-0.50 to -0.05); **p = 0.015** | 8 | SMD = -0.33 (-0.61 to -0.06); **p = 0.017** |
| Number of hospitalizations | 4 | SMD = 0.60 (0.33 to 0.86); **p <0.001** | 4 | SMD = 0.43 (-0.03 to 0.89); p = 0.07 | – | – | 5 | SMD = 0.50 (0.14 to 0.85); **p = 0.006** | 4 | SMD = 0.66 (0.42 to 0.90); **p <0.001** |
| Suicide attempts | 7 | OR = 0.29 (0.20 to 0.44); **p <0.001** | 10 | OR = 0.25 (0.18 to 0.35); **p <0.001** | 13 | OR = 0.25 (0.18 to 0.34); **p <0.001** | 9 | OR = 0.24 (0.16 to 0.37); **p <0.001** | 9 | OR = 0.27 (0.17 to 0.42); **p <0.001** |
| Psychotic features | 5 | OR = 2.01 (0.91 to 4.43); p = 0.08 | 6 | OR = 1.72 (1.10 to 2.69); **p = 0.017** | – | – | 8 | OR = 2.19 (1.45 to 3.31); **p <0.001** | 7 | OR = 2.28 (1.39 to 3.77); **p = 0.001** |
| Hyperthymic temperament | 2 | OR = 2.10 (0.72 to 6.10); p = 0.17 | 4 | OR = 2.36 (1.15 to 4.85); **p = 0.019** | – | – | 3 | OR = 1.90 (0.96 to 3.76); p = 0.06 | 4 | OR = 1.59 (0.89 to 2.83); p = 0.12 |
| Anxiety disorders | 4 | OR = 0.27 (0.14 to 0.52); **p <0.001** | 5 | OR = 0.38 (0.27 to 0.55); **p <0.001** | 5 | OR = 0.36 (0.25 to 0.52); **p <0.001** | 2 | OR = 0.28 (0.14 to 0.57); **p <0.001** | 3 | OR = 0.24 (0.12 to 0.47); **p <0.001** |
| Family history of depression | 3 | OR = 0.67 (0.33 to 1.35); p = 0.26 | 5 | OR = 0.52 (0.33 to 0.83); **p = 0.005** | 5 | OR = 0.55 (0.34 to 0.89); **p = 0.016** | 2 | OR = 0.61 (0.21 to 1.77); p = 0.37 | 4 | OR = 0.59 (0.31 to 1.13); p = 0.11 |

k = number of included studies; CI = confidence interval; OR = odds ratio; SMD = standardized mean difference; – = sensitivity analysis not performed due to the lack of low-quality studies in this item.

Statistically significant estimates are reported in bold.
