# Supplementary material for: Characterizing the clinical profile of mania without major depressive episodes: a systematic review and meta-analysis of factors associated with unipolar mania
Source: Psychol Med. 2023 Apr 5;53(15):7277–86. doi: 10.1017/S0033291723000831 (PMC10719688; doi:10.1017/S0033291723000831)
Supplement: Bartoli et al. supplementary material 3 — Bartoli et al. supplementary material [file S0033291723000831sup003.docx]

**Supplementary File 2. Risk of bias assessment of included studies**

| **Studies** | | **UM and BD**  **comparability** | | **Sample**  **representativeness** | **UM**  **definition** | |
| --- | --- | --- | --- | --- | --- | --- |
|  |  | **Age** | **Illness duration** |  | **Number of manic episodes** | **Observation ≥4 yrs** |
| Aghanwa, 2001 | | **?** | **-** | **+** | **+** | **+** |
| Akarsu et al., 2012 | | **+** | **+** | **+** | **+** | **-** |
| Amamou et al, 2018 | | **-** | **+** | **+** | **+** | **-** |
| Andrade-Nascimento et al., 2011 | | **-** | **-** | **+** | **-** | **+** |
| Angst et al., 2004 | | **-** | **+** | **+** | **-** | **+** |
| Angst et al., 2019 | | **-** | **+** | **+** | **-** | **-** |
| Beesdo et al., 2009 | | **+** | **+** | **-** | **-** | **+** |
| Chang et al., 2022 - GREAT study | | **+** | **+** | **+** | **-** | **-** |
| Dakhlaoui et al, 2008 | | **-** | **+** | **+** | **-** | **+** |
| Gorgulu et al., 2021 | | **+** | **+** | **+** | **+** | **+** |
| Grobler et al., 2014 | | **-** | **?** | **-** | **+** | **-** |
| Grover et al., 2021 | | **?** | **?** | **+** | **+** | **+** |
| Mittal et al., 2013 | | **-** | **+** | **+** | **-** | **-** |
| Perugi et al., 2007 | | **+** | **-** | **+** | **+** | **+** |
| Rajkumar, 2016 | | **+** | **+** | **+** | **-** | **-** |
| Sangha et al., 2022 | | **+** | **?** | **+** | **-** | **-** |
| Sonkurt et al., 2021 | | **+** | **-** | **+** | **+** | **+** |
| Stokes et al., 2020 | France cohort | **-** | **-** | **+** | **+** | **+** |
|  | UK cohort | **+** | **+** | **+** | **+** | **+** |
| Subramanian et al., 2016 | | **+** | **+** | **+** | **-** | **-** |
| Yazici and Çakir, 2012 | | **?** | **?** | **+** | **+** | **+** |
| Yazici et al., 2002 | | **-** | **+** | **+** | **+** | **+** |

UM = unipolar mania; md-BD = bipolar disorder with a manic-depressive course.

**+** = the study meets the quality criterion; **-** = the study does not meet the quality criterion; **?** = unclear.
