# Supplementary material for: Characterizing the clinical profile of mania without major depressive episodes: a systematic review and meta-analysis of factors associated with unipolar mania
Source: Psychol Med. 2023 Apr 5;53(15):7277–86. doi: 10.1017/S0033291723000831 (PMC10719688; doi:10.1017/S0033291723000831)

Supplementary File 3. Association between unipolar mania and male gender

Studies	Estimate (95% C.I.)
Aghanwa, 2001	0.45 (0.18, 1.13)
Akarsu et al, 2012	0.78 (0.23, 2.70)
Andrade-Nascimento et al, 2011	1.71 (0.62, 4.75)
Angst et al, 2004	1.60 (0.72, 3.55)
Angst et al, 2019	1.65 (1.07, 2.55)
Beesdo et al, 2009	2.50 (1.01, 6.16)
Chang et al, 2022	1.29 (0.89, 1.86)
Dakhlaoui et al, 2008	1.16 (0.43, 3.09)
Gorgulu et al, 2021	2.75 (1.11, 6.83)
Grobler et al, 2014	2.54 (1.12, 5.74)
Mittal et al, 2013	1.00 (0.23, 4.43)
Perugi et al, 2007	0.66 (0.23, 1.87)
Rajkumar, 2016	0.47 (0.17, 1.31)
Sangha et al, 2022	2.50 (2.19, 2.84)
Stokes et al, 2020 - France cohort	2.74 (0.83, 9.04)
Yazici and Cakir, 2012	1.81 (0.81, 4.03)
Yazici et al, 2002	1.32 (0.70, 2.48)
	**1.47 (1.11, 1.94)**

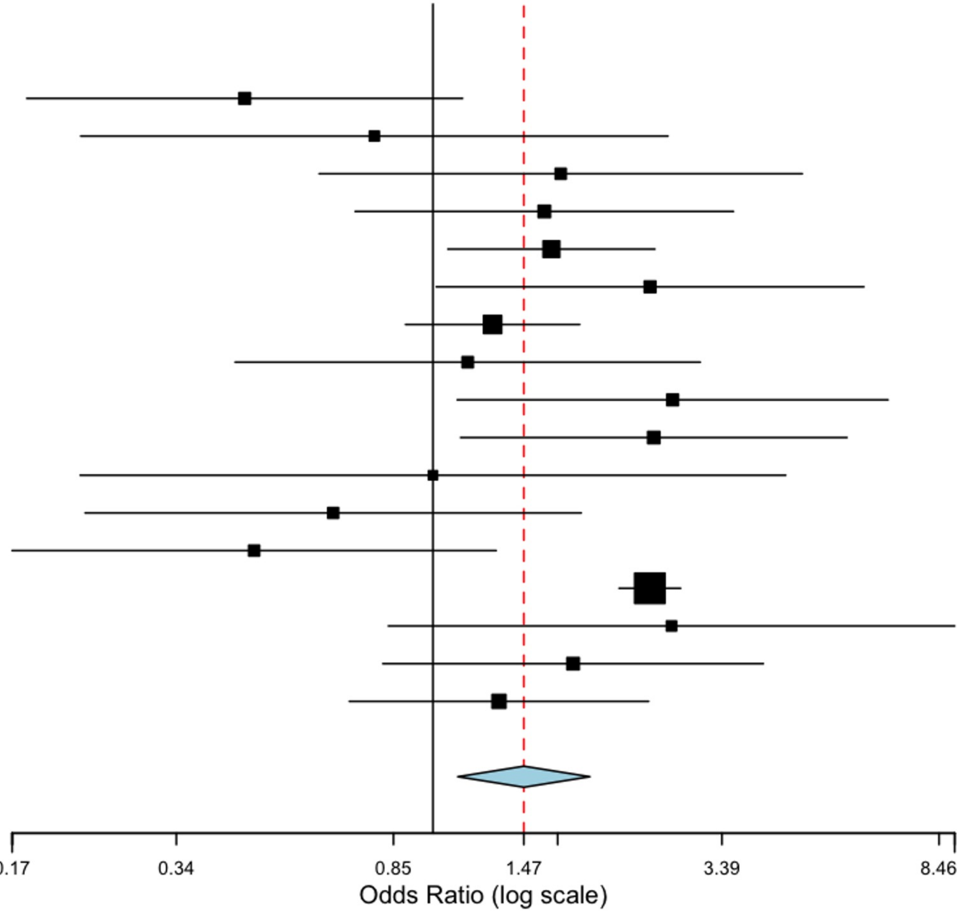

Supplementary File 4. Association between unipolar mania and being married

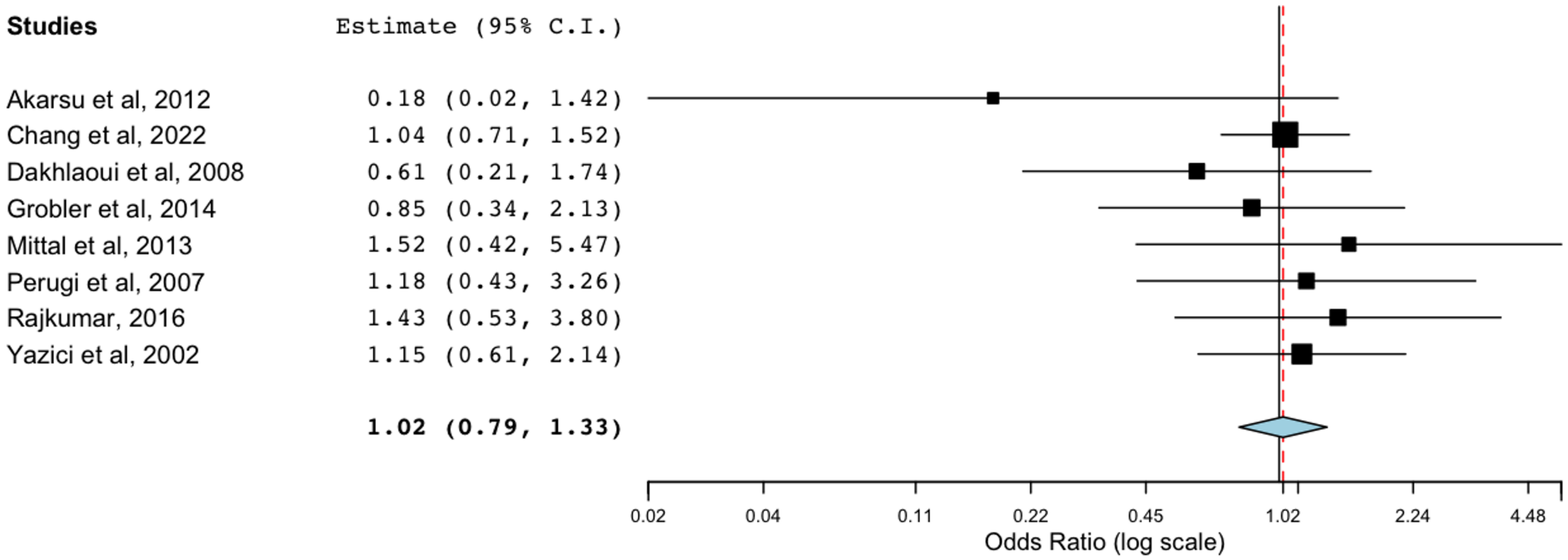

Supplementary File 5. Association between unipolar mania and unemployment

Studies	Estimate (95% C.I.)
Aghanwa, 2001	2.25 (0.89, 5.68)
Amamou et al, 2018	0.95 (0.50, 1.80)
Andrade-Nascimento et al, 2011	1.48 (0.50, 4.39)
Dakhlaoui et al, 2008	0.63 (0.24, 1.69)
Grobler et al, 2014	0.79 (0.33, 1.86)
Mittal et al, 2013	1.64 (0.53, 5.12)
Perugi et al, 2007	0.95 (0.31, 2.84)
Subramanian et al, 2016	0.82 (0.43, 1.56)
	**1.02 (0.75, 1.38)**

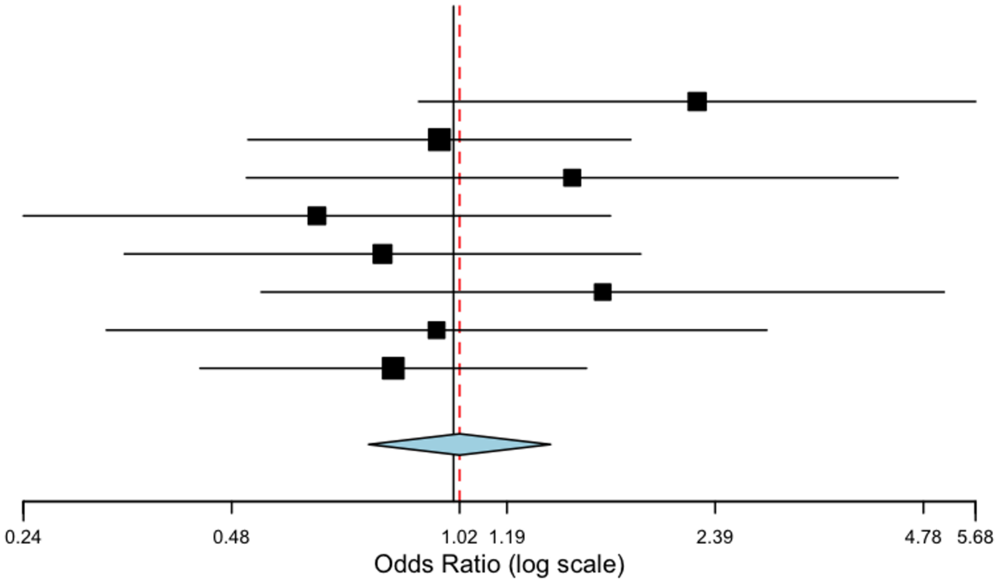

Supplementary File 6. Association between unipolar mania and high-level education

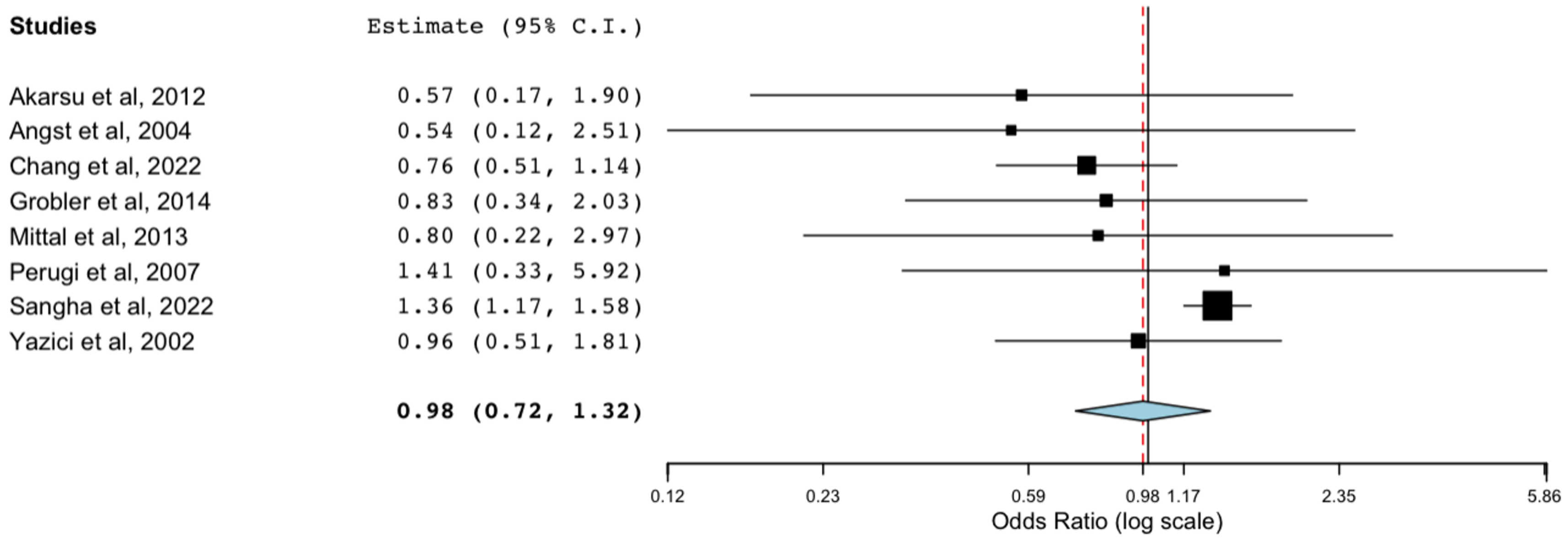

Supplementary File 7. Association between unipolar mania and age at disease onset

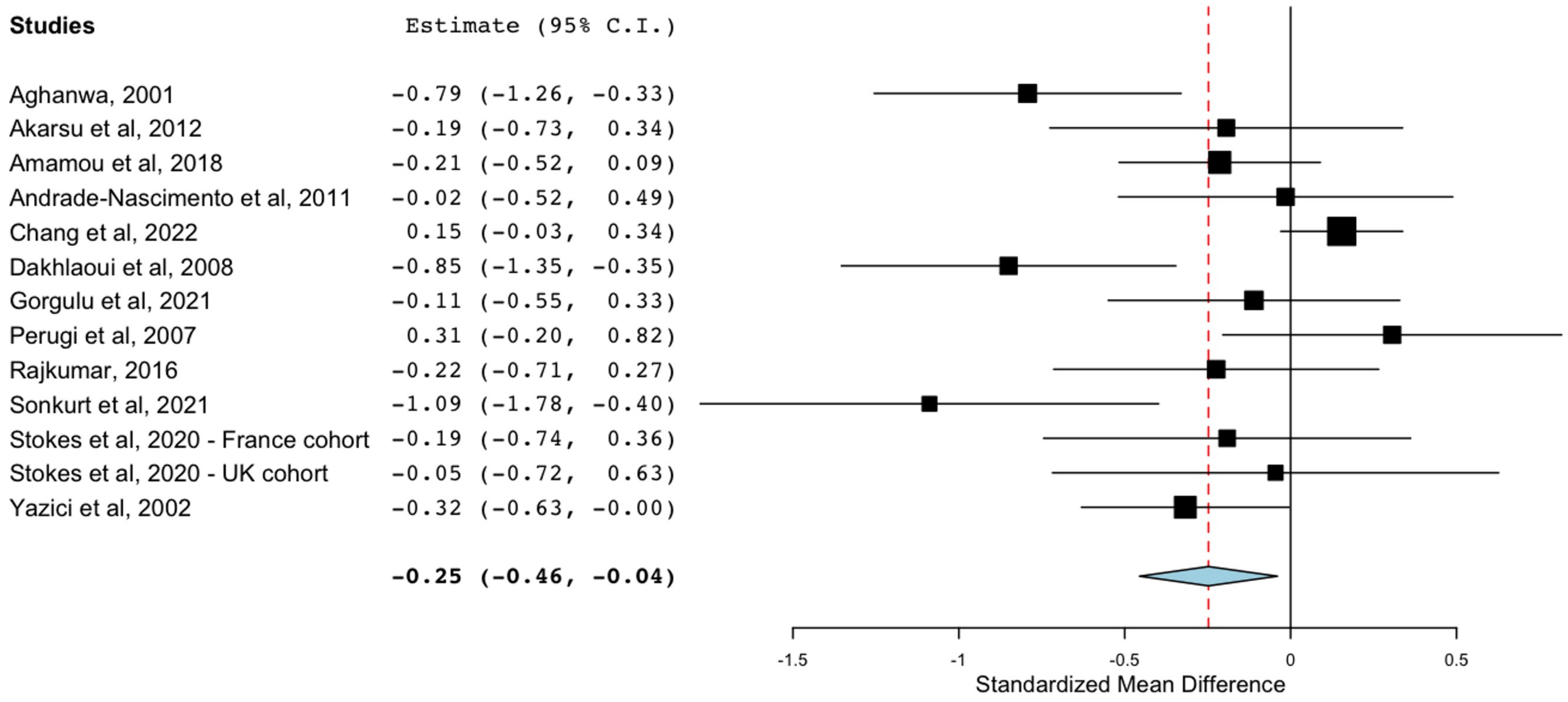

Supplementary File 8. Association between unipolar mania and mood episodes

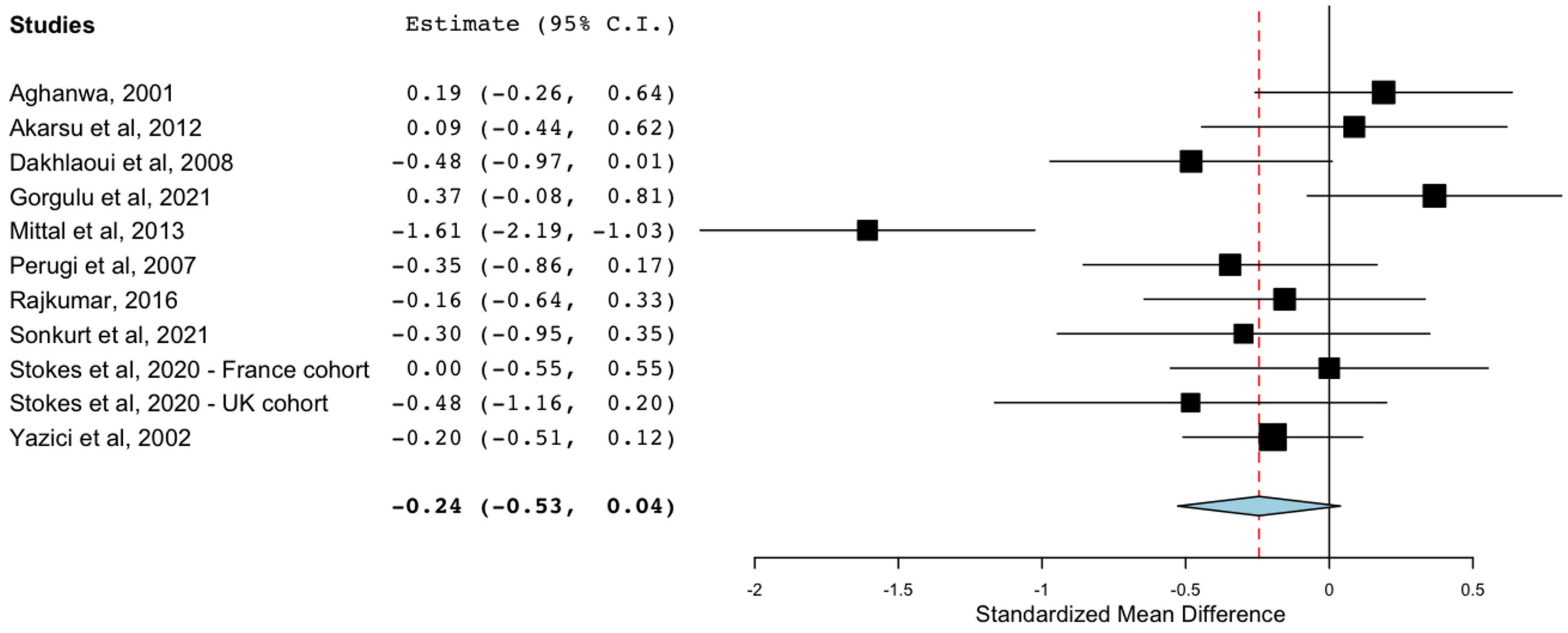

Supplementary File 9. Association between unipolar mania and number of hospitalizations

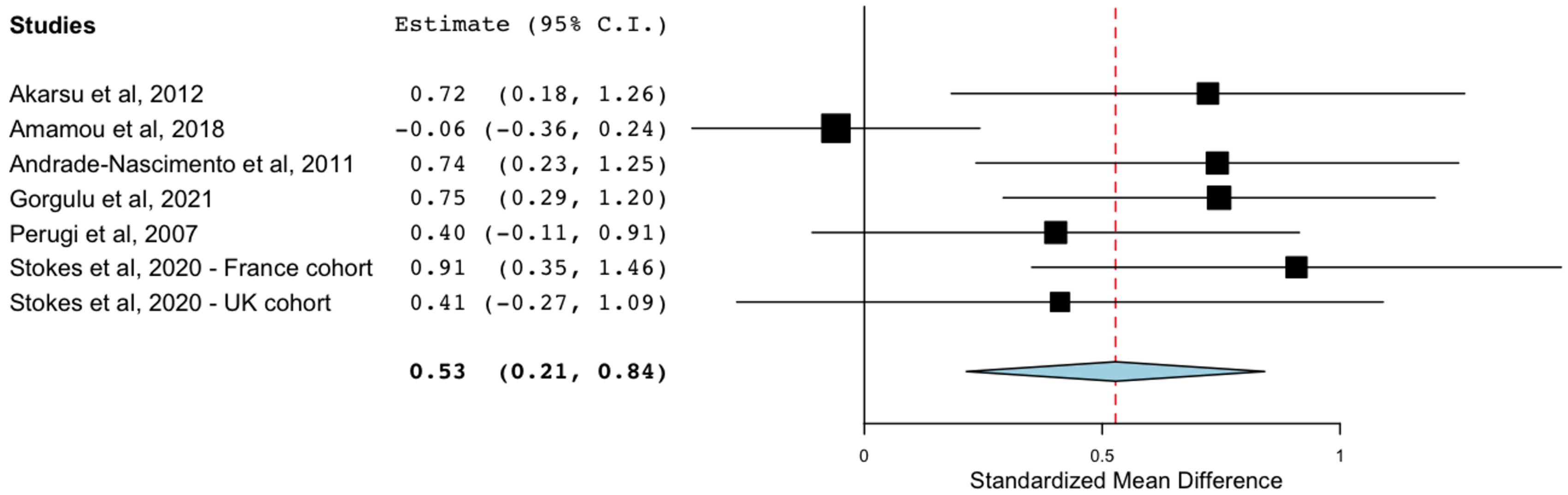

Supplementary File 10. Association between unipolar mania and history of suicide attempts

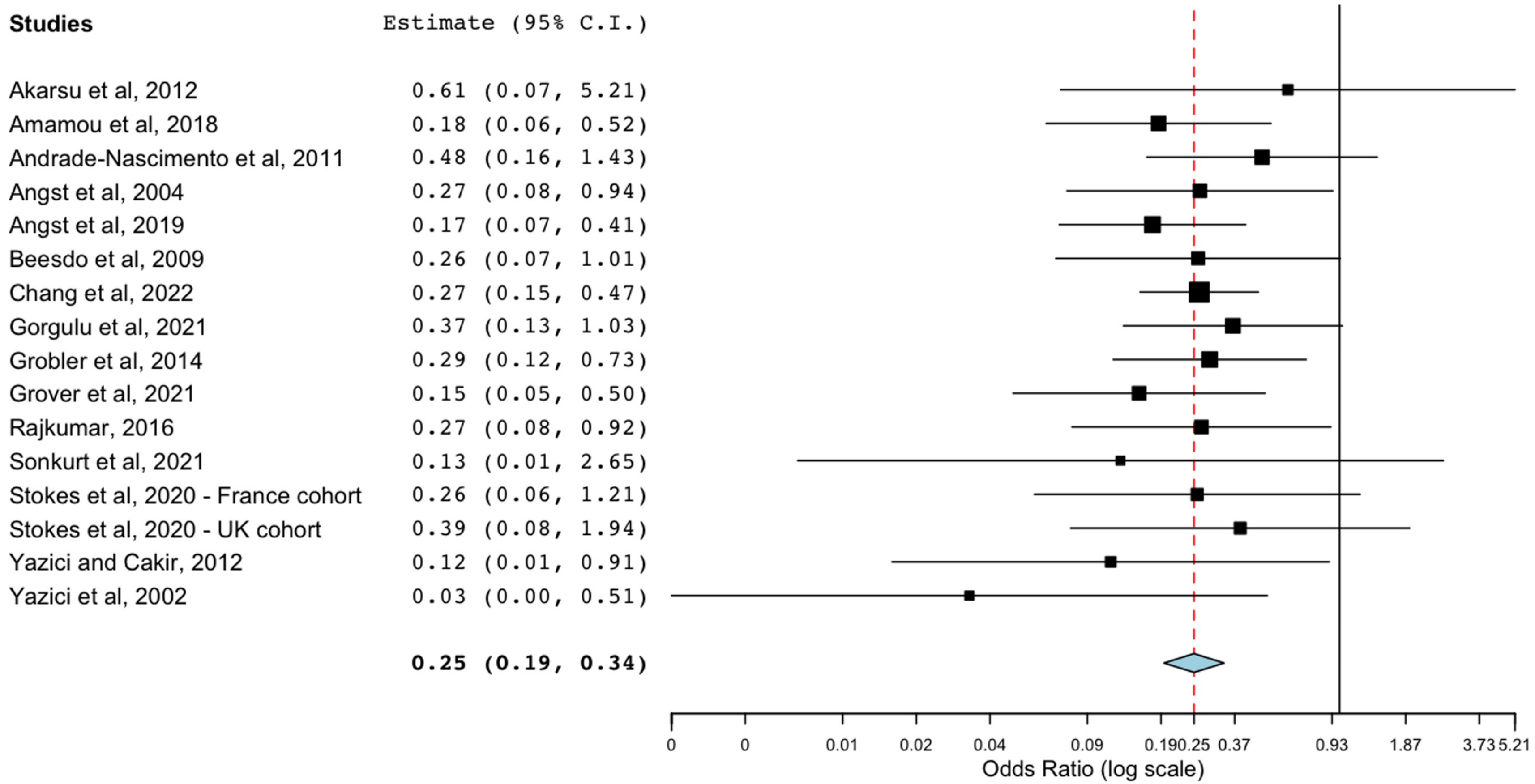

Supplementary File 11. Association between unipolar mania and rapid cycling course

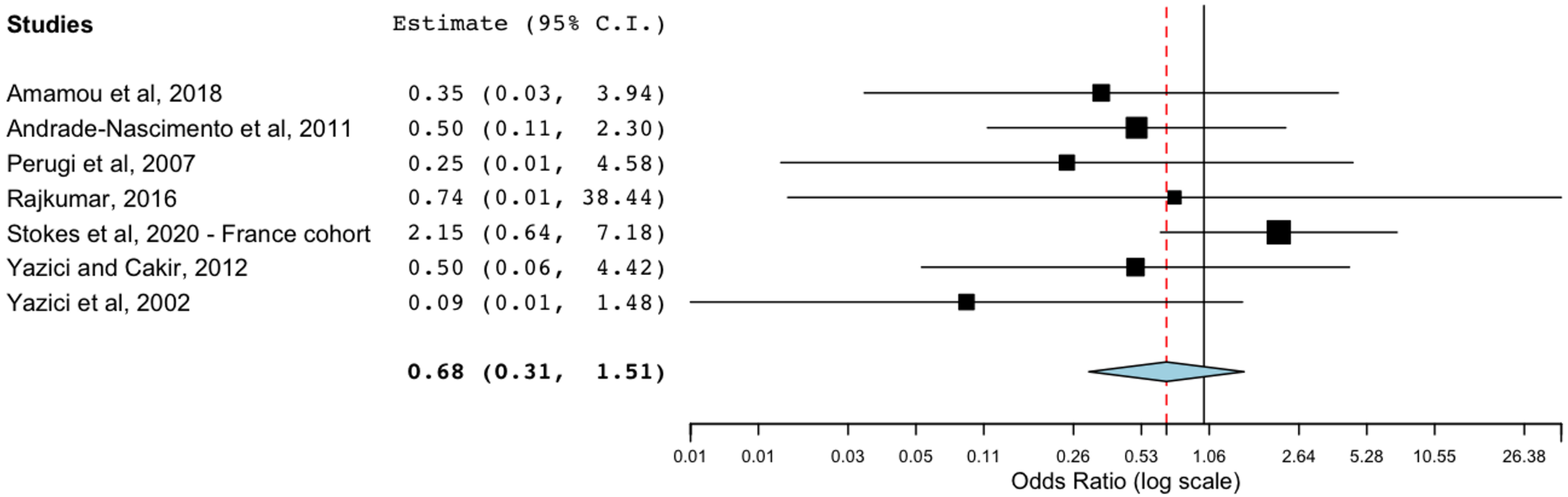

Supplementary File 12. Association between unipolar mania and psychotic features

Studies	Estimate (95% C.I.)
Akarsu et al, 2012	1.01 (0.35, 2.95)
Amamou et al, 2018	1.83 (0.99, 3.37)
Angst et al, 2004	1.10 (0.41, 2.95)
Chang et al, 2022	2.71 (1.67, 4.38)
Grover et al, 2021	2.83 (1.48, 5.43)
Perugi et al, 2007	3.80 (0.80, 17.94)
Sonkurt et al, 2021	5.56 (1.20, 25.71)
Stokes et al, 2020 - France cohort	1.40 (0.38, 5.18)
Stokes et al, 2020 - UK cohort	0.20 (0.02, 2.05)
Yazici and Cakir, 2012	5.17 (1.46, 18.33)
Yazici et al, 2002	2.39 (1.02, 5.61)
	**2.16 (1.55, 3.00)**

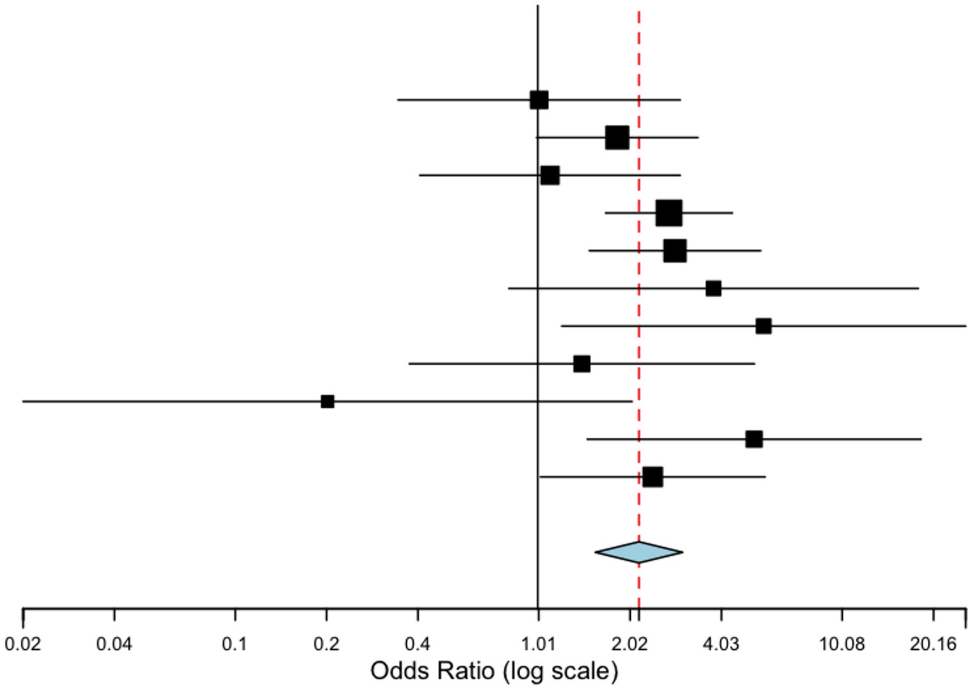

Supplementary File 13. Association between unipolar mania and hyperthymic temperament

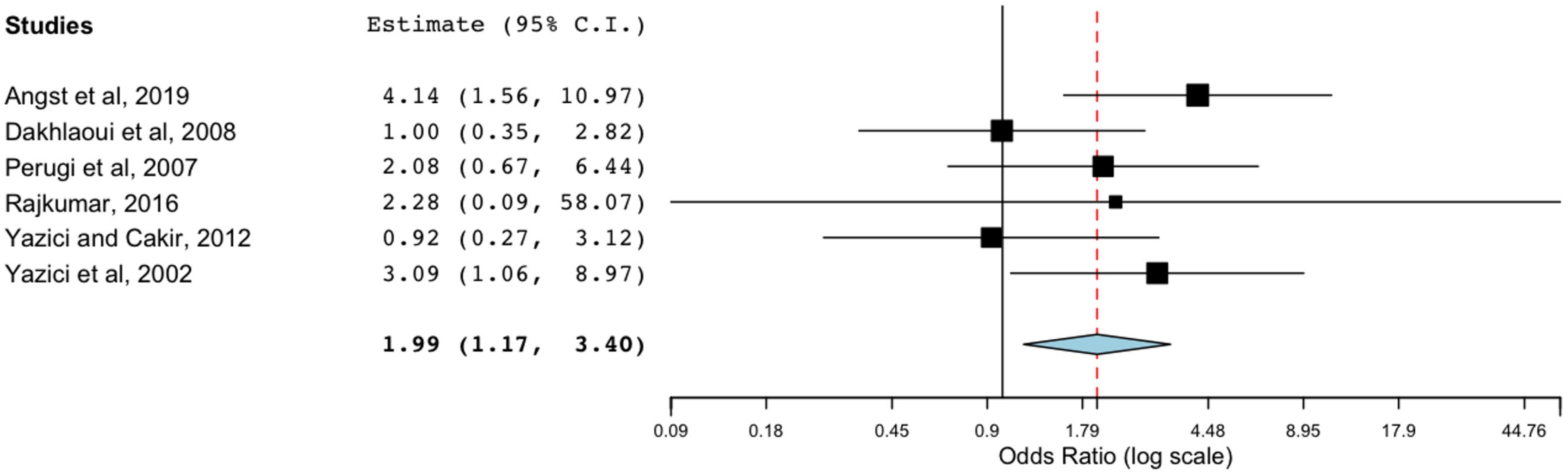

Supplementary File 14. Association between unipolar mania and anxiety disorders

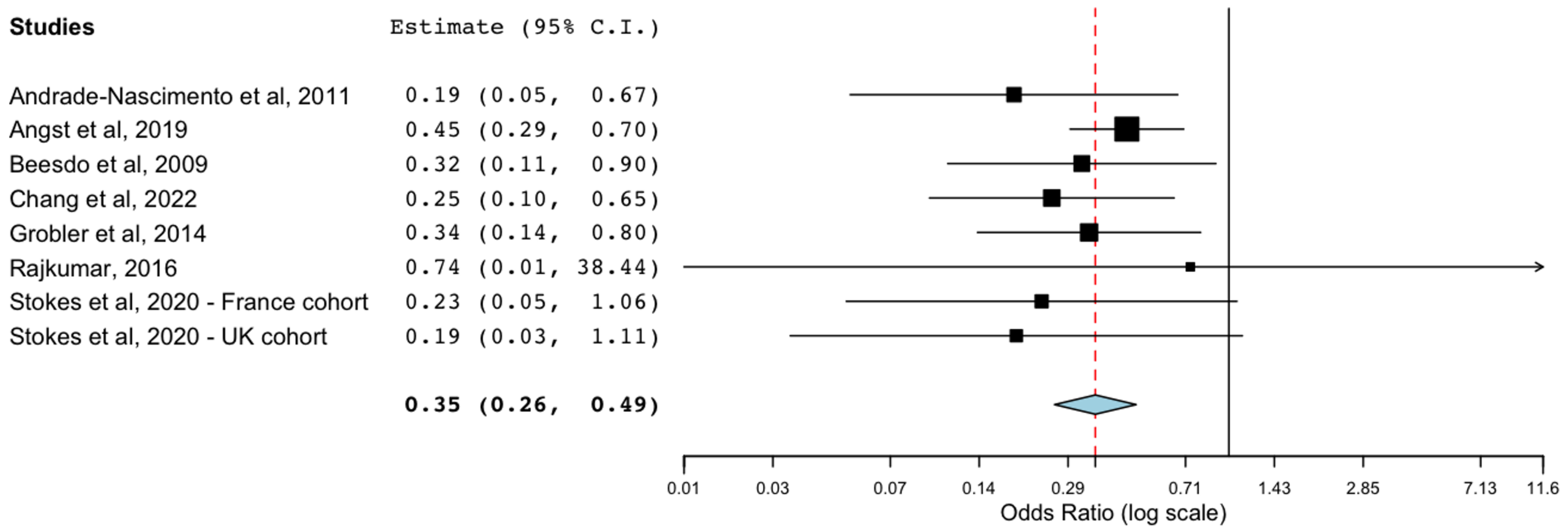

Supplementary File 15. Association between unipolar mania and alcohol use disorders

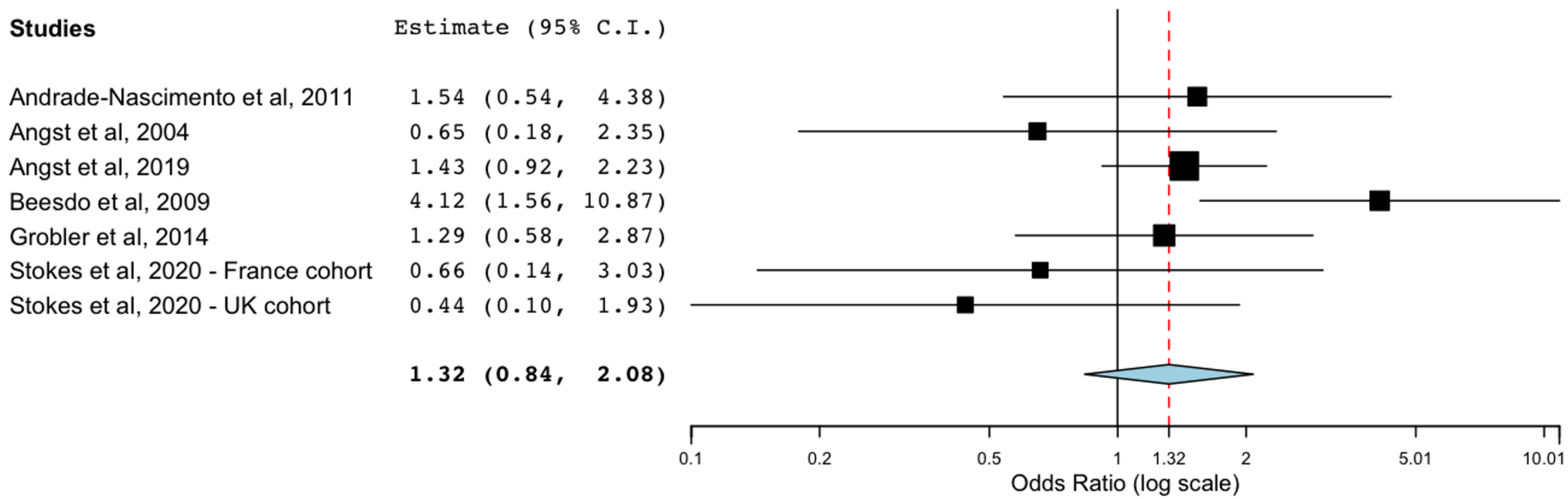

Supplementary File 16. Association between unipolar mania and substance use disorders

Studies	Estimate (95% C.I.)
Andrade-Nascimento et al, 2011	1.42 (0.44, 4.56)
Angst et al, 2004	0.60 (0.03, 11.87)
Angst et al, 2019	0.59 (0.35, 1.01)
Beesdo et al, 2009	1.95 (0.77, 4.91)
Rajkumar, 2016	0.38 (0.11, 1.32)
	**0.86 (0.46, 1.62)**

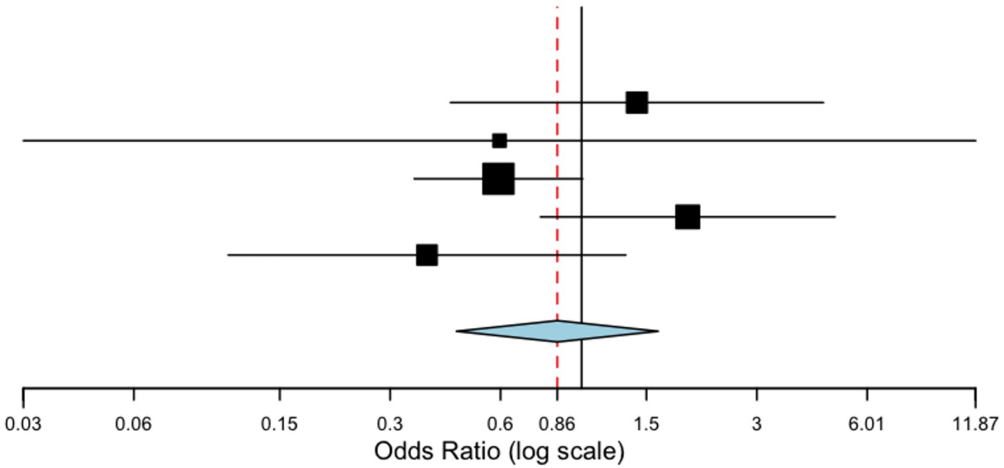

Supplementary File 17. Association between unipolar mania and family history of bipolar disorder

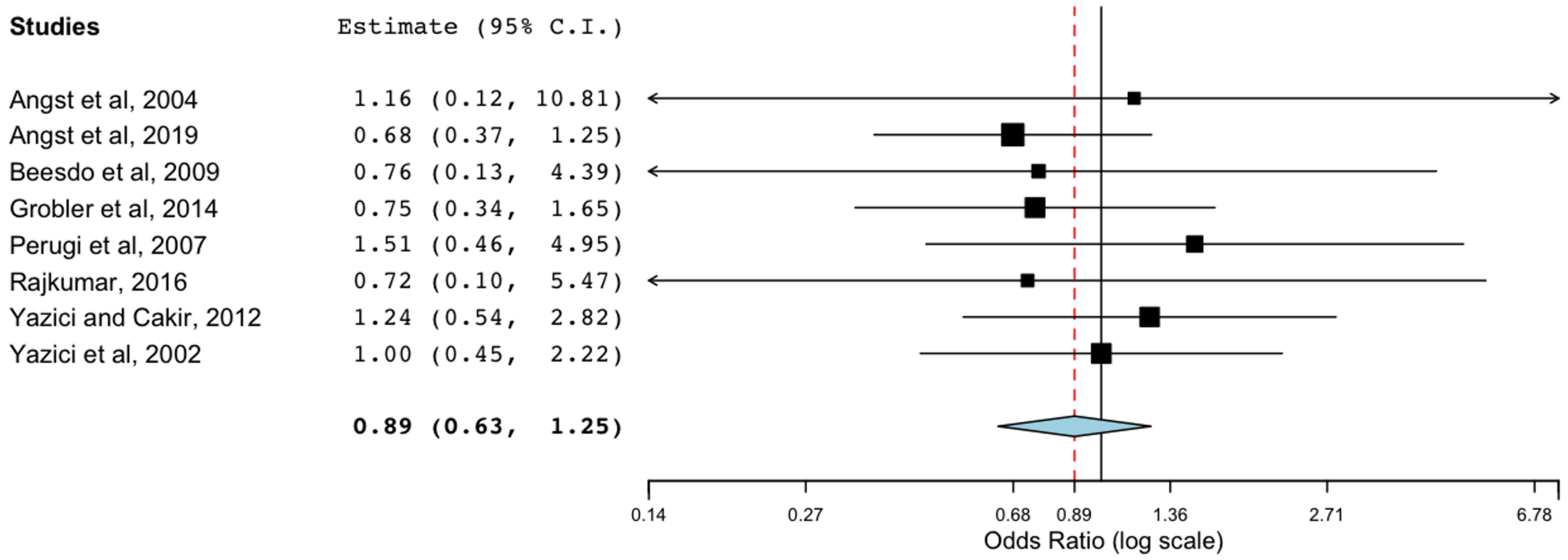

Supplementary File 18. Association between unipolar mania and family history of depression

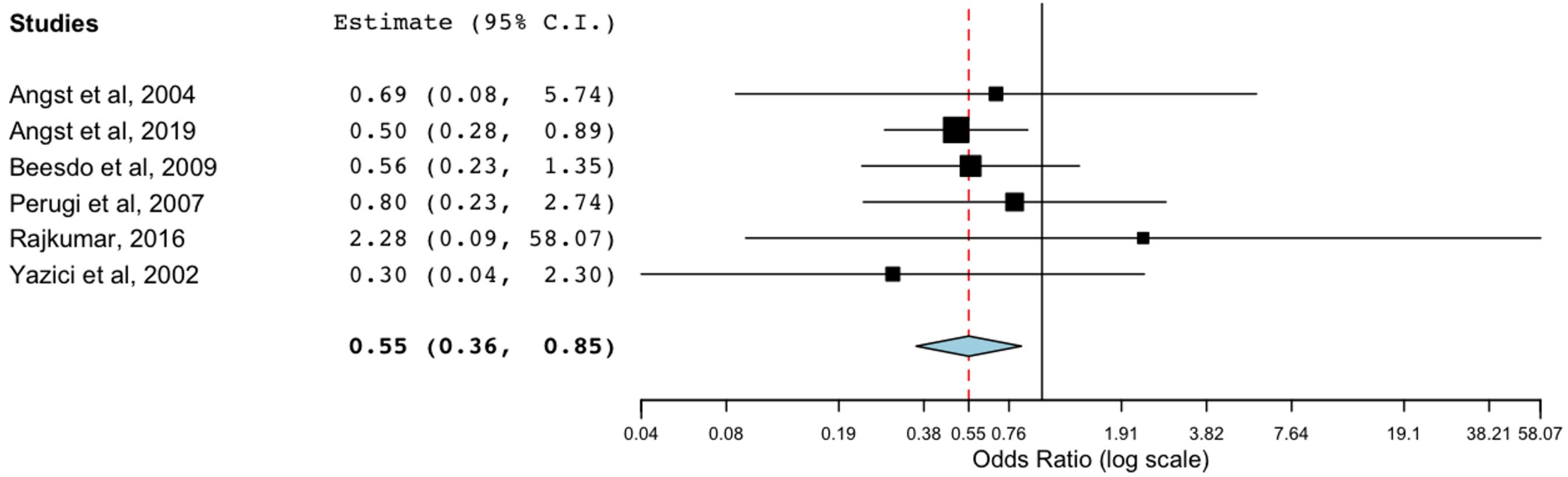

Supplementary File 19. Association between unipolar mania and family history of alcohol use disorders

Studies	Estimate (95% C.I.)
Angst et al, 2019	0.65 (0.27, 1.58)
Beesdo et al, 2009	1.67 (0.65, 4.31)
Grobler et al, 2014	0.82 (0.38, 1.80)
Perugi et al, 2007	0.57 (0.06, 5.08)
Rajkumar, 2016	2.44 (0.69, 8.70)
	**1.04 (0.64, 1.68)**

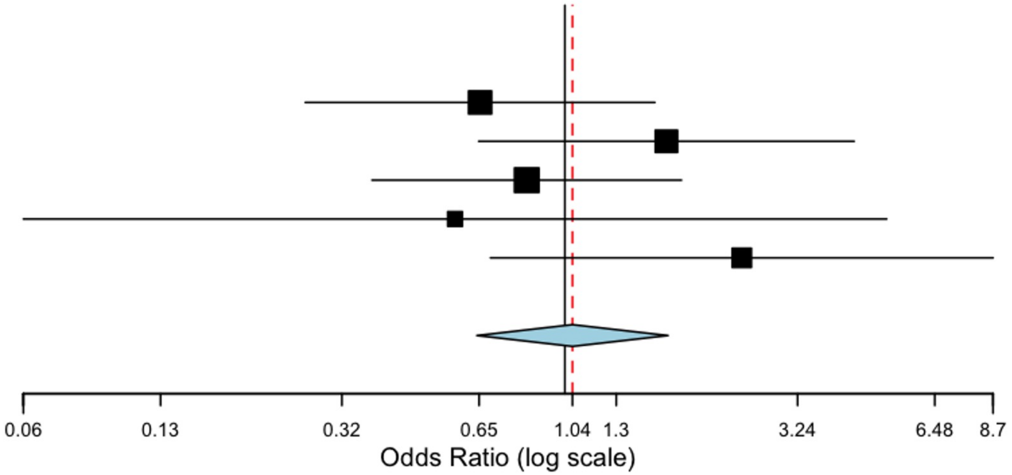

Supplementary File 20. Association between unipolar mania and lithium prescription

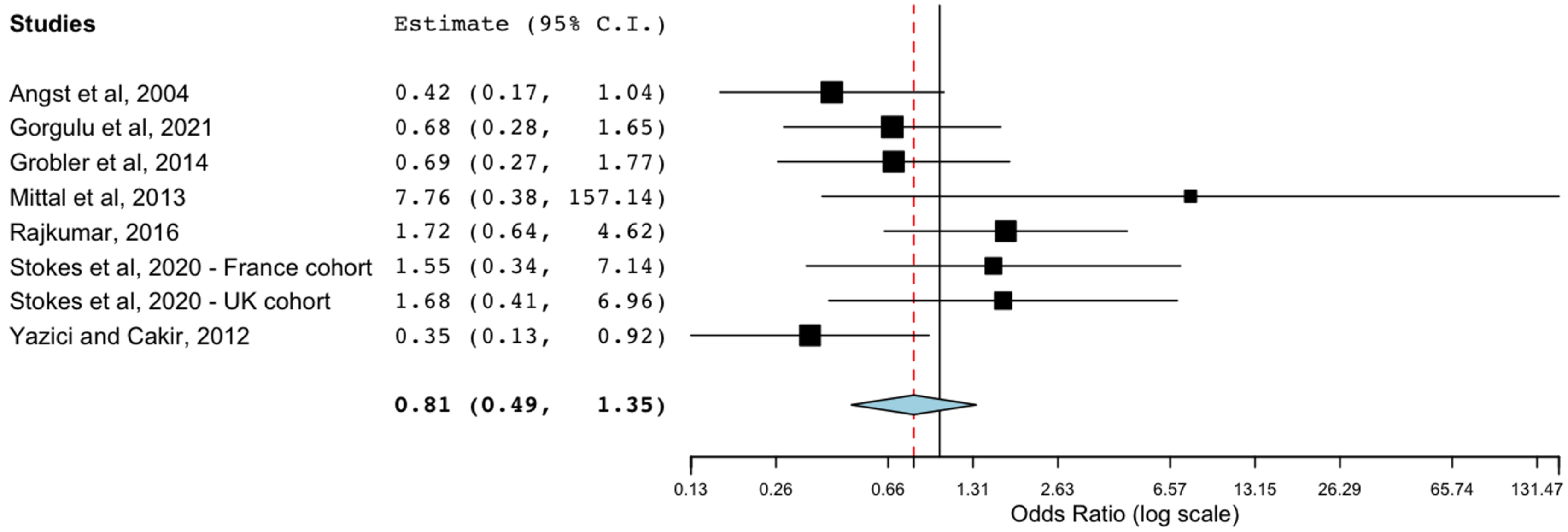

Supplementary File 21. Association between unipolar mania and valproate prescription

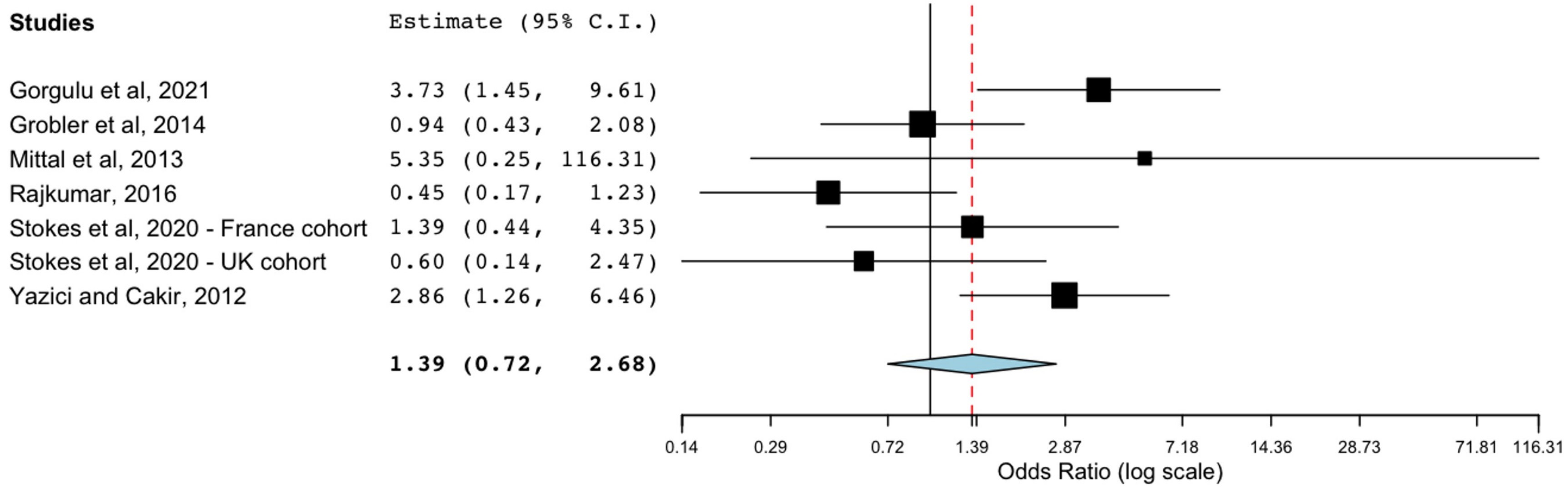

Supplementary File 22. Association between unipolar mania and atypical antipsychotics prescription

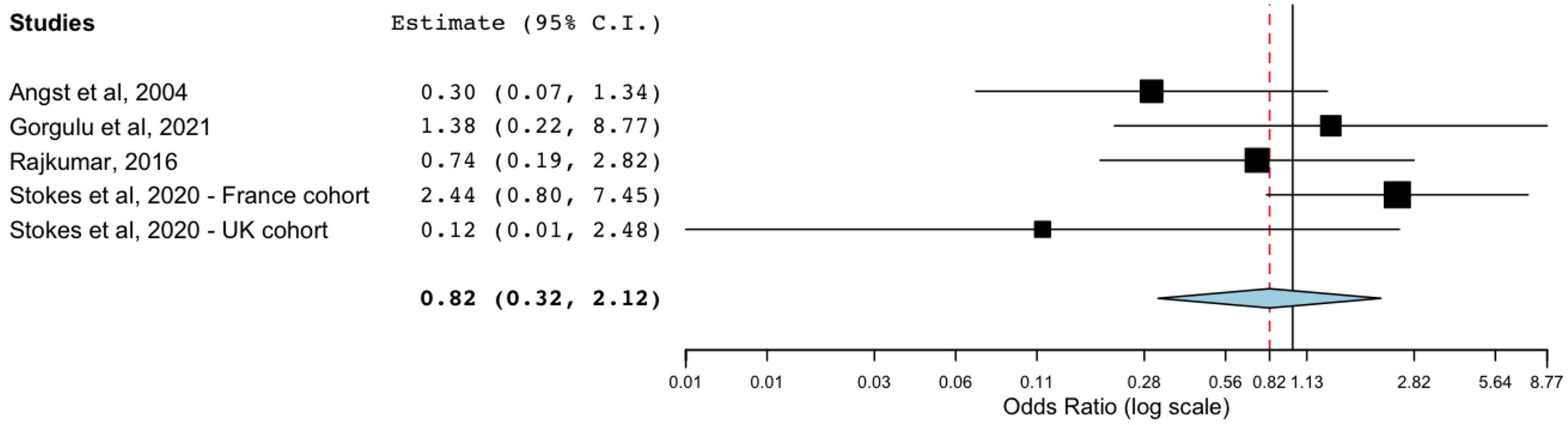

Supplement: Bartoli et al. supplementary material 4 — Bartoli et al. supplementary material [file S0033291723000831sup004.pdf]
